# A new paradigm and computational framework to estimate stop-signal reaction time distributions from the inhibition of complex motor sequences

**DOI:** 10.3389/fncom.2015.00087

**Published:** 2015-07-14

**Authors:** Tobias Teichert, Vincent P. Ferrera

**Affiliations:** ^1^Department of Neuroscience, Columbia UniversityNew York, NY, USA; ^2^Department of Psychiatry, University of PittsburghPittsburgh, PA, USA

**Keywords:** cognitive control, stop signal task, SSRT distribution, motor sequence, race model

## Abstract

Inhibitory control is an important component of executive function that allows organisms to abort emerging behavioral plans or ongoing actions on the fly as new sensory information becomes available. Current models treat inhibitory control as a race between a Go- and a Stop process that may be mediated by partially distinct neural substrates, i.e., the direct and the hyper-direct pathway of the basal ganglia. The fact that finishing times of the Stop process (Stop-Signal Reaction Time, SSRT) cannot be observed directly has precluded a precise comparison of the functional properties that govern the initiation (GoRT) and inhibition (SSRT) of a motor response. To solve this problem, we modified an existing inhibitory paradigm and developed a non-parametric framework to measure the trial-by-trial variability of SSRT. A series of simulations verified that the non-parametric approach is on par with a parametric approach and yields accurate estimates of the entire SSRT distribution from as few as ~750 trials. Our results show that in identical settings, the distribution of SSRT is very similar to the distribution of GoRT albeit somewhat shorter, wider and significantly less right-skewed. The ability to measure the precise shapes of SSRT distributions opens new avenues for research into the functional properties of the hyper-direct pathway that is believed to mediate inhibitory control.

## Introduction

Inhibitory control is an important component of executive function that allows us to stop a planned or ongoing thought and action on the fly as new information becomes available (Logan, 1994; Williams et al., 1999; Boucher et al., 2007; Verbruggen and Logan, 2008). In the early 80s, Logan and colleagues paved the way for a quantitative study of inhibitory control by introducing the concept of an unobservable, inhibitory Stop process (Logan, 1981). In the standard “countermanding” stop signal paradigm (Logan, 1981; Logan et al., 1984) subjects are required to respond as quickly as possible to a Go signal, i.e., to press the right button in response to rightward arrow, or a left button-press for a leftward arrow. On a fraction of the trials, a Stop signal, presented at a random time after the Go signal, instructs subjects to withhold the response. Subjects are worse at inhibiting the pre-potent motor response as the Stop signal is presented closer in time to movement initiation. The horse-race model originally formulated by Logan and Cowan conceptualizes the ability to inhibit the response as a race between a Go- and a Stop process that are triggered by the presentation of the Go and Stop-signal, respectively (Logan, 1981, 1982; Logan et al., 1984; Logan and Cowan, 1984; Stuphorn et al., 2000; Band et al., 2003; Verbruggen and Logan, 2008). If the Go process finishes first, the response will be executed. If the Stop process finishes first, the response will be inhibited.

The success of the model depends on its ability to estimate the duration of the Go and Stop process. The Stop-signal reaction time (*SSRT)* refers to the duration of the stop-process, i.e., the time at which the Stop-process terminates relative to the presentation of the Stop signal. This time is unobservable because no response is emitted on successfully inhibited Stop trials. Current mechanistic implementations of the horse-race model such as the Hanes-Carpenter model (Hanes and Carpenter, 1999), the independent race model (Boucher et al., 2007; Schall and Boucher, 2007) or the special race model (Logan et al., 2014) conceptualize the Stop process as a linear or noisy rise to threshold very similar to models that have successfully been used to model the finishing time of the Go process, i.e., *GoRT*. Other models of inhibitory control such as the interactive race model (Boucher et al., 2007) provide more realistic neural implementations, and allow the Go process and the Stop process to interact. However, for the purposes of the current manuscript, we make the same simplifying assumptions of independence that underlie the original horse-race model (Logan, 1981).

In contrast to the finishing times of the Go process, the finishing times of the Stop process cannot be observed directly and need to be inferred through the absence of a response. Based on the assumption of independence between the Go and Stop-process, the horse-race model enables the estimation of the mean and variance of the SSRT distribution, independent of its precise shape (Logan and Cowan, 1984). So far, however, it has been very challenging to estimate the precise shape of the *SSRT* distribution. This constitutes a significant limitation because *SSRT* distributions may provide important insight into underlying mechanisms of inhibitory control in the same way as *RT* distributions have been used to infer and restrict neural mechanisms of decision-making. In particular, precise estimates of *SSRT* distributions might distinguish subtle functional differences between response initiation and response inhibition that are thought to be mediated by distinct neural substrates, i.e., the direct and hyper-direct cortico-striatal pathway (Aron and Poldrack, 2006; Aron et al., 2007).

In 1990, two labs presented a theoretical method to derive *SSRT* distributions from empirical hazard functions of observed *GoRT* on failed inhibition trials (Colonius, 1990; de Jong et al., 1990). However, hazard functions are notoriously noisy (Luce, 1986) and the required number of stop-signal trials per stop signal delay was estimated at a prohibitively large value of 250,000 (Matzke et al., 2013). More recent approaches have circumvented this problem by parametrizing the *SSRT* distribution (Colonius et al., 2001; Matzke et al., 2013; Logan et al., 2014). This reduces the number of trials that are needed to estimate the distribution, albeit at the cost of restricting the possible shapes that can be recovered.

Here we present an alternative approach to this problem by using a different type of inhibitory task that conveys more information about SSRT on each trial. We based our task on the complex movement inhibition task by Logan (1982). In his task professional typists were asked to type a word or a sentence until a stop-signal instructed them to stop typing. Interestingly, Logan concluded that subjects were equally likely to stop the complex motor sequence at any point in time during the word or sentence and that SSRT was independent of whether or not the stop-signal appeared before or after typing began. This suggests that such typing tasks measure the same concept of stop-signal reaction time measured in the countermanding task. However, in contrast to the countermanding task, the complex movement inhibition task has the potential to provide more information about the occurrence of the stop-signal on a trial-by-trial basis. In particular, it provides a hard lower estimate (the stop process must have finished after last key was typed), and it provides a soft upper estimate (the stop-process must have finished before the next key press would usually have occurred). If the interval between key-presses is short and predictable, each trial can provide a rather narrow window for SSRT. However, even for the skilled typists in Logan's study, the interval between key-presses was on average 200 ms thus leaving a relatively large window that does not provide a substantial increase in the amount of information per trial. Based on these considerations we modified Logan's complex movement inhibition task. To increase the rate of button presses we asked subjects to press any sequence of their choosing on the keyboard as fast as possible. Using this instruction even subjects without special training in typing achieved mean inter-button press interevals around 30 ms. This value is small enough to provide a substantial increase in information that can be gained on each trial. We refer to this task as the Motor-Sequence Inhibition task (SeqIn).

The current study focuses the novel theoretical framework and analysis technique that we developed to extract information about the speed of inhibitory control from the SeqIn task or similar tasks that require subjects to stop an ongoing motor sequence with discrete behavioral output such as typing. In particular, the study highlights the feasibility of a novel deconvolution method to extract non-parametric estimates of entire SSRT distributions. The study also provides a detailed description of the subjects' behavior in the SeqIn task to understand and rule out potential confounds.

## Methods

### Ethics statement

All participants provided written signed informed consent after explanation of study procedures. Experiments and study protocol were approved by the Institutional Review Boards of Columbia University and New York State Psychiatric Institute.

### SeqIn task

We developed a novel *SSRT* task to facilitate the parameter-free estimation of *SSRT* distributions with a reasonable number of trials. In this new task subjects perform an unconstrained, self-ordered motor sequence (button-pressing on a keyboard at a rate of ~30 per second) until a stop-signal instructs them to stop. The task is referred to as the Motor-Sequence Inhibition task (SeqIn). The time of the last button press relative to the stop-signal can be measured on each trial. Under the assumption of independence of the Go and Stop process, the distribution of the observed last button presses can be understood as the convolution of the distribution of *SSRT* with a distribution X that can be estimated from the data (see below). In the classical stop-signal or countermanding task, a single go and stop process race against each other. Here, the task can be thought of as a sequence of go processes (one for each key press) terminated by a single stop process.

#### Behavioral task

In the SeqIn task subjects placed the fingers of their left and right hands on a keyboard as they would for typing. Each finger was assigned one particular key (left pinky: “*a”*; left ring finger: *“s”*; left middle finger: *“d”*; left index finger: *“f”*; right index finger *“j,”* right middle finger: *“k,”* right ring finger: *“l,”* right pinky: *“;”*). A pure tone auditory cue (880 Hz) instructed the subject to immediately start pressing as many buttons as possible using any sequence of finger-movements. Subjects were discouraged from pressing multiple buttons at the same time, or holding down a button for a prolonged period of time. Otherwise, subjects were free to use any sequence of their choosing. In the current study we did not record the identity of the button presses. Hence, we cannot quantify the type of sequences the subjects chose. However, from observation and own experience, the subjects chose regular repeating patterns of finger-movements. After a random interval (see below) during which the subjects continued pressing buttons, the same auditory cue instructed them to stop as fast as possible. Hence, the same stimulus served as the “go-signal” during transition to the response period as well as the “stop-signal” during transition to the no-response period. An additional visual cue reminded subjects of the current state of the task. This cue was green during the response period and red during the no-response period.

**Figure 1:**
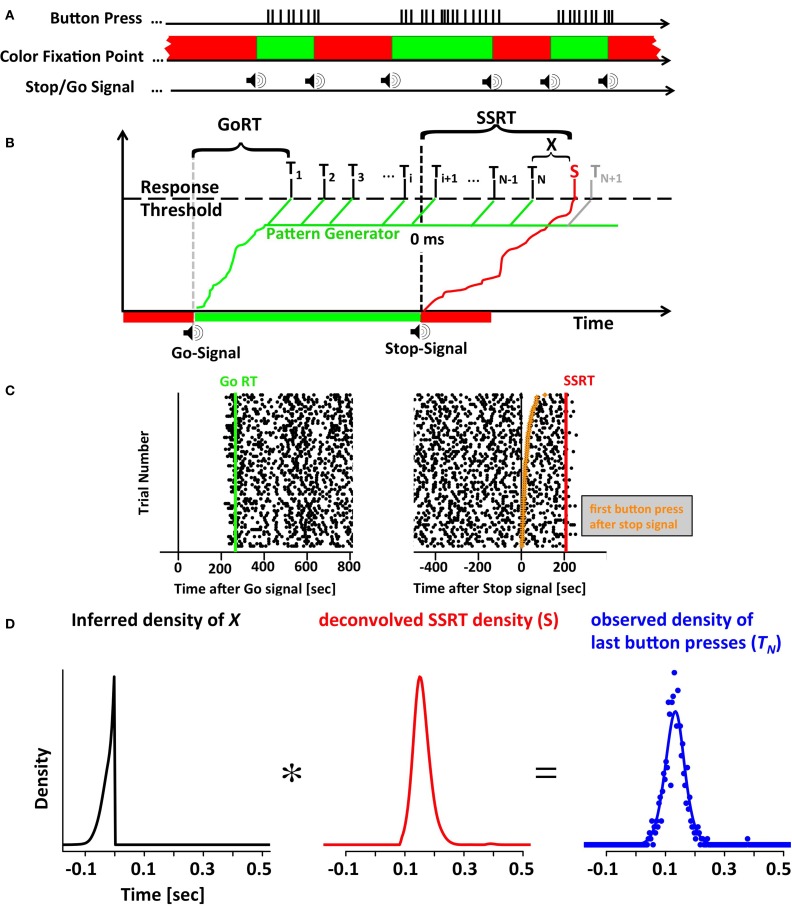
**The motor-sequence inhibition paradigm (SeqIn). (A)** In the SeqIn paradigm subjects place all fingers of their left and right hand (except the two thumbs) on a computer keyboard as they would for typing. During the response period they are instructed to press as many buttons as possible using whichever sequence of finger-movements they choose. Subjects are discouraged from pressing multiple buttons at the same time, or holding down a button for a prolonged period of time. After an exponentially distributed waiting period, a 880 Hz tone instructed them to start button pressing as quickly as possible. After another exponentially distributed response period, the same tone instructed subjects to stop the motor sequence as soon as possible. The color of the fixation spot was green in the Go- and red in the Stop-period. **(B)** One potential theoretical framework for the SeqIn paradigm: the multi-process race model. Following the Go signal, a Go processes starts developing toward a threshold that activates a pattern generator that generates sequences of motor commands. A response is assumed to occur whenever a motor command reaches the response threshold (*T_i_*). GoRT was defined as the time of the first button press after the presentation of the Go-signal (*T*_1_). The total number of observed button presses is defined as *N*. Hence, the time of the last button press relative to the Stop-Signal is *T_N_*. The Stop signal triggers the emergence of a Stop process that also develops toward the response threshold. Once the Stop process reaches the threshold at time *S*, all upcoming planned button presses (*N* + 1, *N* + 2, …) will be inhibited. The stop signal reaction time can be determined as the sum of the last button press *T_N_* and an estimate of *X*, the difference between *T_N_* and *S*. The estimate of X uses the distribution of observed button-press intervals to predict the distribution of *T_N+1_* (see Methods). **(C)** Example data set with times of button presses aligned to the Go signal (left panel) and the Stop signal (right panel). Each trial starts with the presentation of the go-signal, lasts throughout the go-period as well as the subsequent stop-period. Trial number was sorted according to the time of the first button press after the Stop signal (orange points in right panel). **(D)** The distribution of last button presses *T_N_* can be viewed as the convolution of the distribution of *X* and the unobservable density of the Stop process *S*. Since the distributions of *X* and *T_N_* can be either be directly measured or inferred from the data, the *SSRT* distribution can be estimated via deconvolution.

A trial was defined as a single go-period followed by a single stop-period. One run consisted of a series of 25 go and 25 stop periods that were presented in immediate succession with no time between them (Figure 1A). The minimum duration of each go and stop period was 1.5 s. In addition to the 1.5 s baseline, we added a random interval that was drawn from a truncated exponential distribution with a maximum value of 3.5 s and a time constant of 0.572 s. The total duration of each period varied between 1.5 and 5 s.

#### Training

The current study aimed to explore the possibilities of the SeqIn task in the best possible circumstances. Hence, particular care was taken to recruit subjects that had already performed other experiments and were known to be reliable psychophysical subjects. All subjects performed at least 3 runs of 25 trials of the SeqIn task on a day prior to the start of the main experiment.

#### Setup

The experiments were performed on MacBook Pro Laptop computers. The task was programmed and executed with Matlab2009a using routines from Psychtoolbox-3 (Kleiner et al., 2007). Auditory go- and stop-signals were presented through a pair of commercial headphones (Bose) plugged into the 3.5 mm stereo jack. Experiments were conducted in a dark experimental room. Viewing distance was 24 inches. The two experimental computers had 13 and 17-inch screens, giving rise to slightly different viewing angles. Individual subjects were tested consistently in one of the two setups. Screen resolution was set to 1280 by 800 pixels.

#### Subjects

The participants were 6 experienced human psychophysics subjects (2 female) of age 22–42, including the first author. All participants provided written informed consent after explanation of study procedures.

### The deconvolution algorithm

The horse-race model treats response inhibition as a race between a Go- and a Stop process. If the Go process finishes first, the response is executed. If the Stop process finishes first, the response is successfully inhibited. We have expanded this framework to include multiple Go processes to account for the multiple button presses in the SeqIn task. Figure 1B visualizes one particular mechanistic implementation of this expanded framework. It is based on the assumption that each go signal triggers a Go process that activates a pattern generator. The pattern generator sends out individual Go-processes that trigger a response as soon as they reach a fixed threshold. The stop-signal triggers the initiation of a Stop process that inhibits the execution of additional button presses as soon as it reaches threshold. The considerations outlined below are non-parametric and independent of this particular implementation. If the last button press was observed at time *t*, then the stop signal must have finished at some unknown later point in time *t+x*. In the following we will derive a method to estimate the distribution of *x*.

Let *T_i_ i* = 1, 2, 3, … be a series of random variables that represent the time of the ith button press. By default we report *T_i_ relative to the time of the stop signal* (Figure 1C). However, the *T_i_*'s can also be reported relative to the time of the go signal. GoRT can be defined as the time of the first button press *T*_1_ relative to the go signal. Let *N* be an integer-valued random variable that represents the number of the last executed button press before the Stop process finishes and prevents further responses. Hence, *T_N_* represents the time of the last button press. Let Δ*_i_* be a set of random variables that describes the time between two adjacent button presses:

(1) $$\Delta_{i}=T_{i}-T_{i-1}\;\forall 1<i\leq N$$

For simplicity we assume that all Δ*_i_* are independent and have the same distribution:

(2) $$F_{\Delta_{i}}=F_{\Delta }\;\forall i\in \mathbb{N}$$

Let *S* be a random variable that represents the time at which the Stop process finishes. Then *X* = *S* − *T_N_* represents the time between the last button press and the termination of the Stop process. Conversely, we can define *S* as the sum of *T_N_* and *X*. Under the assumption that *T_N_* and *X* are independent, the distribution of *S* corresponds to the convolution of the distributions of *T_N_* and *X*.

(3) $$S=T_{N}*X$$

Based on Equation (3) we can estimate the unobservable distribution of *S* by deconvolving *X* from *T_N_*. The distribution of *X* can be estimated in two different ways. Both approaches are based on the assumption that *S* and *T_i_* are independent. The first approach is more intuitive and starts with the trivial observation that *X* cannot be measured explicitly because *S* is not observable. However, based on the existing trial data we can simulate trials in a scenario where *S* is know. To that aim define a suitable variable *S′* as the simulated time at which the stop-process terminates. For this simulated time-point *S′* we can now pretend that all button presses after *S′* did not occur, as would have been the case if *S′* had been the actual time at which the stop signal terminated. In the simplest case, we can define *S′* = 0 ms for all trials, i.e., pretend that the stop-process terminates instantaneously at the exact time the stop signal is presented. In analogy to our definition of *N* as the number of the last button press before the termination of the *actual* stop-process, we can now define *M* as the number of the last button press before the termination of the *simulated* stop process. In this scenario it is possible to explicitly calculate *X′* as the difference between *S′* and *T_M_*. As long as both *S* and *S′* are independent of *T_i_*, the distribution of *X′* will be identical to the unobservable distribution of *X*. Note that the definition of *M* is useful only if *S′* is smaller than *S*. Otherwise, we can't be sure that *M* was actually the last button press before *S′*: the termination of the actual stop process at time *S* might have inhibited later button presses that could have occurred before *S′*. In practice this is an easy requirement to meet by only using *S′* values before the presentation of the stop signal, i.e., *S*′ = 0. Of course it is similarly important to chose *S′* such that *S*′ > *T*_1_. To give subjects time to establish a steady pressing routine, we use an even stricter criterion and ensure that *S′* is always at least 500 ms larger than *T*_1_. For each value of *S′*, we get one estimate of *X′* for each recorded trial. Given the large number of trials recorded and the large range of possible values for *S′* it is easy to estimate *X′* and hence *X* with great precision.

The second approach is based on the density of inter-button press intervals *f*_Δ_. To that aim we first assume that Δ_*N*+1_ is known and equal to a fixed value *u*. Due to the independence of *S* and *T_i_, S* is equally likely to have terminated at any point after *T_N_* and before *T_N_*+u. Hence, *X* is uniformly distributed over the interval from 0 to *u*, with a density equal to *1/u*. This is true for any value of *u*. We can now integrate across all possible values *u* weighted by the likelihood that Δ_*N*+1_ is equal to *u*:

(4) $$\begin{array}{l} F_{X}\left(t\right)=P\left(X\leq t\right)\\ \;=\int_{0}^{\infty }\left(\frac{\min\left(t,u\right)}{u}\right)f_{\Delta }du\\ \;=\int_{0}^{t}f_{\Delta }\left(u\right)du+\int_{t}^{\infty }\frac{t}{u}f_{\Delta }du\\ \;=F_{\Delta }\left(t\right)+t\int_{t}^{\infty }\frac{1}{u}f_{\Delta }du \end{array}$$

We can solve and differentiate Equation (4) numerically to obtain an estimate of the density of *X*. Due to the large number of observed inter-button press intervals, the distribution of *X* can be estimated very accurately.

We confirmed that both methods yield numerically highly similar estimates of the distribution of *X*. The first approach provides a simple intuition and can be interpreted as the distribution of last button presses under the assumption of an instantaneous stop signal. The second approach is appealing because of its more stringent mathematical derivation.

After estimating the density of *X*, we can use it to recover S by deconvolving *X* from *T_N_* (Figure 1D). Convolutions cannot always be inverted, in particular when the convolution kernel has no power in a frequency band that the original signal does. This was not a concern in the current case, as the convolution kernel *X* has a broad power spectrum (note the sharp edge in Figure 1D, left panel). To further stabilize the deconvolution process, the distribution of *T_N_* was smoothed with a Gaussian kernel with a standard deviation of 16 ms. This reduced the noise in the estimate of the distribution of *T_N_*. In the noise-free case, the result of the deconvolution should recover the *SSRT* density function *S* smoothed with the same 16 ms Gaussian kernel. However, in the noisy case, the result of the deconvolution needs to be processed further to ensure that the outcome fulfills the requirements of a density function. In particular, the imaginary component of the deconvolution was set to zero. We then integrated the resulting raw density over time to recover a raw distribution function. The lower time bound was defined as the latest time at which the raw distribution function was negative. Negative raw distribution values occurred due to small ripples of the raw density function that tended to be present at the left tail of the raw density function. All density values below or equal to the lower bound time were set to zero. Any residual negative density values above the lower bound time were also set to zero. Independent of the above operations, density values below 90 ms and above 550 ms were set to zero. Finally, the resulting function was normalized to a sum of one. Neither of these operations changed the overall shape of the distribution but were necessary in order to calculate expected value, variance, and skew.

In addition to the model-free non-parametric deconvolution algorithm outlined above, it is also possible to use a parametric approach. Here we convolved an exponentially modified Gaussian distribution (ex-Gauss) with the distribution of X, and then adjusted the parameters of the ex-Gauss such that the result matched the observed times of the last button press, *T_N_*. Similarly we adjusted the parameters of the ex-Gauss to match the time of the first button press, T_1_, or *GoRT*. In both cases, the fitting process used a maximum likelihood estimation technique. To avoid infinite log-likelihood values during the fitting process we added a lapse rate of 1% evenly spread over all time-bins. The parametric approach has the advantage that it does not depend on the smoothing with the 16 ms Gaussian kernel and hence recovers the parameters of the actual *SSRT* and *GoRT* distribution rather than their smoothed version. This comes at the cost of restricting the possible range of shapes that can be recovered.

To verify the deconvolution approach, we used the same method to recover the known *GoRT* distribution (*T*_1_). To that aim, we first convolved the GoRT distribution with the distribution of X. This was done empirically: for each trial *n* we drew a random sample *x(n)* from the distribution of *X*. This sample was subtracted from the GoRT of this trial:

CRT(n)=GoRT(n)−x(n)

*CRT* (Convolved Reaction Time) provides an estimate of how the RT distribution would look like if it could not be measured explicitly, but rather if it would have to be inferred in the same way as SSRT, i.e., by the absence of some other discrete event. Most importantly, the distribution of CRT is an empirical convolution of GoRT and X. Hence, we can use the deconvolution method to recover the original GoRT distribution. This comparison provides a test for the accuracy of the deconvolution approach.

All computations were performed with the statistical software-package R (R Development Core Team, 2009). Densities were represented in 300 four-millisecond bins ranging from −400 to +800 ms. Trials with extreme *GoRT* or *T_N_* values were excluded from the analysis. However, rejection criteria were very liberal such that for 5330 trials in the seven data sets only 15 trials were rejected. Trials were rejected if their GoRT was below 90 ms or if the time of the last button press *T_N_* happened before the stop signal. The upper bounds for *GoRT* and *T_N_* were defined as their median plus eight times the standard deviation. The same criteria were used for the simulated data sets.

### Analysis of mean SSRT independent of deconvolution approach

In addition to the deconvolution approach described above, it is possible to estimate the mean SSRT from the data using a simpler method that does not rely on the deconvolution method. This simpler method does not provide an estimate of the entire distribution, but provides reliable estimates of mean SSRT from as little as 75 trials. The method relies on the definition *X* = *S* − *T_N_*, which can be reformulated to *S* = *T_N_* + *X*. Hence, *E*[*S*] = *E*[*T_N_*] + *E*[*X*]. We can estimate *E*[*T_N_*] as the mean of the observed *T_N_*, and *E[X]* from the distribution of *X* that was derived in Equation (4). The distribution of *X* depends on the inter-button press intervals which remained rather constant throughout the experiment. Hence, to a first approximation *E[X]* is a constant and *E[S]* is equal to the mean of *T_N_* plus a constant.

### Analysis of recovered densities

The deconvolution algorithm provides estimates of SSRT and GoRT density. To visualize the shape of the distributions independent of inter-subject differences in mean and variance we subtracted out the mean and normalized the standard deviation to one. The normalization was performed on the labels of the time-bins: Let *t* be the sequence of values that denotes the center of each density bin. We then re-assigned the labels for each time bin by subtracting the mean and dividing by the standard deviation: t′ = (t-E[t])/sqrt(V[t]). We then used linear interpolation to estimate density values on a regular grid ranging from −8 to 8 standard deviations and a step size of 0.1. The identical approach was used to normalize the time-axes of the distribution functions.

For visualization purposes we estimated the average normalized density over all subjects. To that aim we averaged the individual normalized *density* functions for each bin across all subjects. This approach was aimed at extracting the shape of the distribution while removing potential differences in mean and width. The normalized *distribution* functions were averaged using Vincent averaging. To that aim we picked a sequence of probabilities ranging from 0.001 to 0.999 in steps of 0.001. For each of these values we used linear interpolation to estimate the corresponding quantile. The mean of these quantile values defined the average distribution function. The resulting distribution function was defined on an irregular grid of time-values. We then used linear interpolation to estimate the distribution function on the regular default grid described above (from -8 to 8 with a step size of 0.1).

The averaging process described above was aimed at removing differences in mean and standard deviation to highlight potential differences in shape. We took another step to re-introduce the information regarding mean and standard deviation while maintaining the information about the shape of the distributions. To that aim we first re-scaled the default time-axis with the square root of the mean of the individual variances. Then we then added the mean of the individual means to the rescaled time-axis. The entire transformation allowed us to give a precise estimate of the average shape as well as their width and position.

## Results

We developed a novel theoretical framework and analysis technique to facilitate the parameter-free estimation of *SSRT* distributions from the inhibition of an unconstrained motor sequence (button-pressing on a keyboard, see Figure 1 and Methods). Six subjects recorded one or more data sets of 525 or more trials (mean 761 trials, maximum 1575 trials) of the SeqIn task. One subject recorded two data sets in two distinct settings for a total of 7 full data sets. Each trial corresponded to one go- and one stop-period and provided the times of all button presses including the first button press after the go-signal, as well as the last button press following the presentation of the stop-signal.

### General properties of behavior in SeqIn task

Before moving on to the calculation of the entire SSRT distribution, we describe some basic properties of the button press patterns in the SeqIn task. In particular, we addressed three main points: (1) What is the rate of button presses that the subjects achieved? (2) Is there any evidence that the subjects used stereotyped motor patterns? (3) Are parts of the sequence ballistic or can the motor sequence be interrupted with equal probability at any point in time? (4) Are there any systematic changes of SSRT or GoRT over time? All of these analyses are important to put the results of the following deconvolution analysis into perspective. The first and third points allow us to quantify the amount of information that can be gained from a single SeqIn trial. The second point helps us gauge the cognitive effort involved in maintaining the motor sequences: it has been suggested that the generation of truly random sequences may require significant cognitive effort. Hence, the use of a stereotyped and presumably automated response pattern supports the idea that the subjects were free to focus on starting and stopping demands of the task without being distracted by the task of maintaining the motor sequence. The fourth point will help us understand the potential contribution of systematic changes in SSRT on the results of the deconvolution. Note that for these analyses we estimate mean SSRT using a more robust method that does not depend on the deconvolution approach (see Methods).

**Figure 2:**
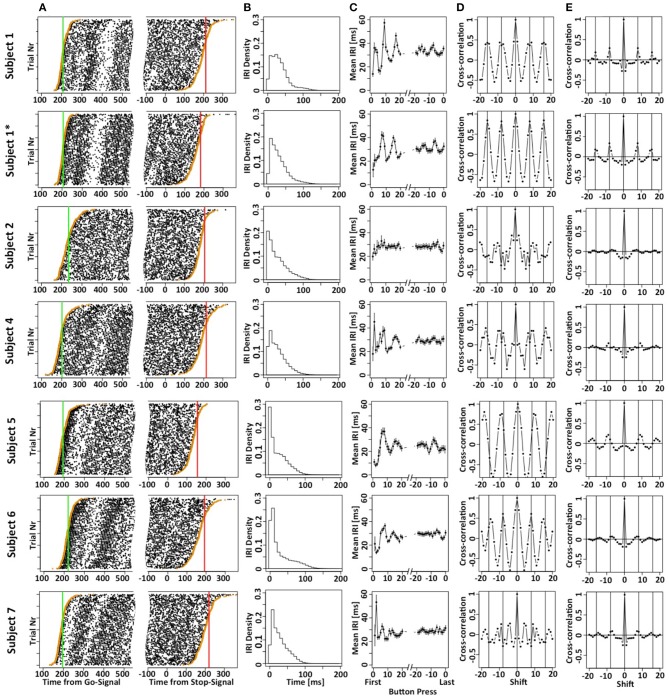
**Motor-sequence patterns the SeqIn task**. **(A)** Left panel: times of button presses (black dots) aligned to the go-signal. Each line corresponds to one trial. Trials are sorted according to the time of the first button press (orange dots). The green line corresponds to the mean GoRT. Right panel: times of button presses aligned to the Stop Signal. Trials are sorted according to the time of the last button press (orange dots). The red line corresponds to the estimated mean SSRT that is calculated as the sum of the mean of the last button press plus an estimate of the mean of *X*. **(B)** Density of the inter-button-press intervals (*IRI* or Δ*_i_*). **(C)** Mean *IRI* as a function of button press number in a trial, either relative to the first button press (left side of panel) or the last button press in a trial (right side of panel). **(D)** Cross correlation of the mean *IRIs*. Vertical lines are spaced at multiples of 8, the number of fingers that subjects can use to generate the sequences. The cross correlation includes the first 40 IRIs. **(E)** Mean of single-trial cross correlation after subtracting the mean IRI that gives rise to the patterns in **(D)**. The data excludes the first 16 IRIs and extends up to IRI 80. Hence, these cross-correlograms indicate rhythmic motor sequences that are not phase-locked to response onset. The star refers to the fact that subject 1 contributed two data sets. The first data set is indicated as S1, the second one is indicated as S1^*^.

Figure 2A shows the button press responses of the subjects in a raster plot format either aligned to the Go (left panels) or Stop signal (right panels). By sorting the order of the trials, it is possible to appreciate the cumulative distribution functions of the times of the first and last button presses. Figure 2B plots the density of the inter-response intervals (IRI) averaged across all instances, and Figure 2C plots the mean IRI as a function of button press number in each motor sequence. Subjects achieved mean IRIs around 30 ms corresponding to ~34 button presses per second. It is important to note that the high rate of button-presses is essential for our purposes to increase the information content of each trial. The shorter the average IRI, the more information each trial carries about the occurrence of the unobservable stop-process. If the last button press occurred at 200 ms after the stop-signal, then the stop-process must have finished some time between 200 ms and the time at which the next press would have occurred if it had not been inhibited. If subjects press a button on average once every 100 ms, this restriction is less strict than if they press on average once every 10 ms. Hence, it was essential to allow subjects to use as many response buttons as possible to increase the rate of button presses and decrease the average IRI. We have also collected preliminary data when subjects were only allowed to use a total of two or four fingers. These preliminary studies revealed similar mean SSRT values, but required a larger number of trials.

The systematic variation of mean IRI with button press number indicates that most subjects used stereotyped mini-sequences. We followed up on this assumption by calculating the cross-correlation of the mean IRIs for the first 40 button presses. If subjects use stereotyped mini-sequences, we would expect cyclic modulation of inter-button-response intervals. Given that a sequence would likely consist of all 8 fingers from both hands (subjects were not allowed to use their thumbs), we would expect the inter-button press intervals to be auto-correlated with a lag of 8 button-presses. This is indeed what we observed in most of the subjects (Figure 2D). We further tested if the stereotyped mini-sequences extend beyond the first couple of button presses. To that aim, we excluded the first 16 IRIs, expanded the analysis throughout IRI number 80, and calculated IRI cross-correlations after subtracting out the mean IRI (Figure 2E). As expected, the cross-correlation values are significantly smaller due to the use of single trial IRIs, rather than mean IRIs. Nevertheless, the analysis indicates cyclic patterns for most of the subjects. Overall, our analysis suggests the use of a stereotyped and presumably automated response pattern. This indicates that the subjects were free to focus on starting and stopping demands of the task without being distracted by the task of maintaining the motor sequence.

**Figure 3:**
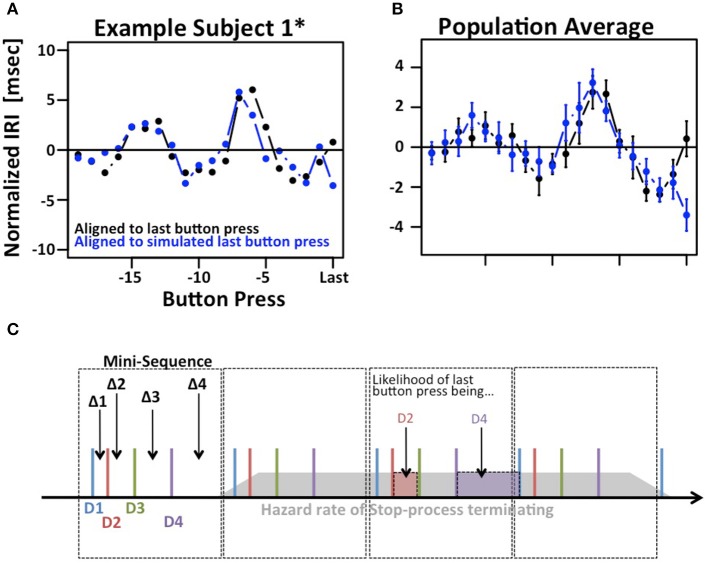
**Modulation of IRIs prior to last button press**. **(A)** Example of systematic modulation of IRIs aligned to the last button press in each trial, i.e., the last button press that could not be inhibited by the stop-process (black). Almost identical modulations can be observed when IRIs are aligned to the last button press prior to a random event, in this case the presentation of the stop-signal (blue). **(B)** Same plot averaged across the population. **(C)** Putative explanation of both phenomena: Subjects employ regular “mini-sequences” consisting of either 4 or 8 button presses. Cartoon example depicts a regularly repeating mini-sequence consisting of 4 button presses executed with digits *D1* trough *D4* thus giving rise to IRIs *Δ1* trough *Δ4*. The assumption is that the stop-process is equally likely to terminate at any point during the sequence (gray polygon). The likelihood of the last IRI being sampled e.g., from *Δ1* is identical to the likelihood of the last button press being *D2*. This in turn is proportional to the average time between *D2* and *D3*, i.e., *Δ2*. Hence, the mix of *Δi* s that contribute to the last IRI prior to a random event is not uniform, but proportional to *Δ(i+1)*s. The star refers to the fact that subject 1 contributed two data sets. The first data set is indicated as S1, the second one is indicated as S1^*^.

The results depicted in Figure 2C show a systematic variation when IRIs are aligned to the last button press of each trial (right hand side of panels 2C). Given the existence of mini-sequences outlined above these variations likely arise because the stop-process is more or less likely to finish between different button-presses in the mini-sequence. For example, it might be possible that the motor sequence can only be stopped at the end of a mini-sequence. This explanation would assume that the hazard rate for the stop-process changes at different times in the mini-sequence. If this were the case it would reduce the amount if information gained from a single trial of the SeqIn task. However, there is an alternative explanation for the observed variations that is consistent with the idea that the stop-process is equally likely to finish at any point in time during the mini-sequence: Figure 3 shows that we can observe almost identical variations when IRIs are aligned to the last button press prior to a random event that is by definition equally likely to occur at any point during the mini-sequence. In summary, the systematic variations of IRI arise because the stop-process is more or less likely to terminate after a particular button press of the mini-sequence. However, this variation in likelihood is explained by the average wait-time for the next button press in the mini-sequence, not a variation of the hazard rate at different points of the sequence (Figure 3C). Hence, the systematic variations of IRI in Figure 2C are not only consistent with, but the logical consequence of the fact that the stop-process is equally likely to finish at any point in time of the ongoing mini-sequences. This result is consistent with Logan's finding in the complex movement inhibition task that typing can be aborted with equal probability at any point in a word or sentence.

The analysis in Figure 3 reveals another potentially interesting phenomenon: The biggest difference between the two IRI sequences occurs on the very last IRI. This finding suggests that the time of the last button press occurs ~3 ms later than expected. While this effect is small and does not reach significance after correcting for multiple comparisons, it may be an indication that the stop-process is not an all-or-nothing process as suggested by the strict assumptions of the independent race model. Rather, the finding would be more in line with the physiologically more realistic interactive race-model (Boucher et al., 2007).

**Figure 4:**
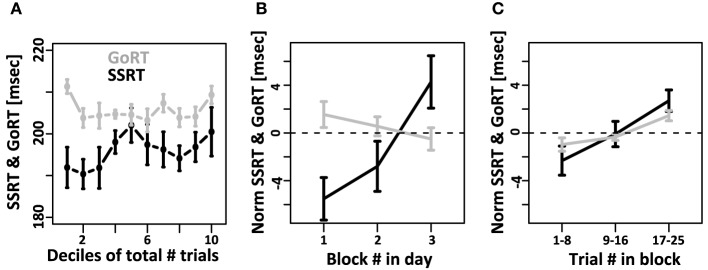
**Systematic changes of SSRT and GoRT over time**. **(A)** For each subject, the total number of trials over all recording sessions was divided into 10 equally sized deciles. GoRT did not significantly vary as a function decile. For SSRT there was a significant effect. However, this effect was carried exclusively by one data set. This data set can be considered an outlier for technical reasons: instead of being collected in small sessions of three blocks divided over multiple days, it was collected in two long sessions. Excluding this data set yields flat SSRT estimates independent of time in the experiment. **(B)** On each recording day, subjects performed at least 3 blocks of 25 trials each. Changes of SSRT and RT are plotted as a function of block number in the recording session. For SSRT, we observe a significant slowdown over the course of each day. In contrast, no such effect is observed for GoRT. **(C)** Slow-down of SSRT (black) and GoRT (gray) as a function of trial number within each block of 25 trials. For both variables there is a significant slow-down over the course of a block. The slowdown seems more pronounced for SSRT, but this difference is not significant.

We then tested if mean SSRT and mean GoRT changed systematically over the course of the experiment. To that aim we divided the data from each subject into 10 equally sized bins (deciles) and calculated mean GoRT and SSRT for each bin (Figure 4A). GoRT did not significantly vary as a function of decile. For SSRT, however, there was a significant effect. Follow-up analyses revealed that this effect depended on one specific data set (data set 1 of subject 1). This data set can be considered an outlier for technical reasons: instead of being collected in small sessions of three blocks divided over multiple days/experimental sessions, it was collected in two long sessions. Excluding this data set yields flat SSRT estimates independent of time in the experiment.

We further tested if mean GoRT and mean SSRT remain stable over the course of each recording session. To that aim we calculated GoRT and SSRT for the first, second and third block of each recording day separately. Figure 4B shows a pronounced and significant SSRT slowdown over the course of the three blocks. In contrast, there is no such effect for GoRT. If at all, there is a small, albeit non-significant, effect in the opposite direction. We also tested if we can identify a slow-down of SSRT even within individual blocks of 25 trials. To that aim we divided each block into 3 sub-sets of trials. Between the first and third sub-set of trials, we observed a significant SSRT slowdown on the order of 4 ms (Figure 4C). It is noteworthy that we also observed a significant within-block slowdown of GoRT. The slowdown of GoRT is less pronounced, but not significantly different from the one we observed for SSRT.

After establishing the general properties of the button-press patterns in the task, we then turned to the deconvolution algorithm to recover the entire SSRT distribution from the times of the last button press (see Figure 1 and Methods). However, in order to assure that the findings from the real data were valid, we first performed simulations where we used the deconvolution approach to recover a known SSRT distribution.

### SSRT deconvolution of simulated data

A series of simulations was conducted to estimate the accuracy of the deconvolution algorithm to recover the true distribution of the SSRT. For each simulated trial, a *GoRT* as well as a *SSRT* were drawn from ex-Gauss distributions with known parameters (see below). In addition, a series of values was drawn from the distribution of inter-response intervals (see Figure 1) observed across all subjects in the study. *T*_1_ was defined as GoRT and the subsequent *T_i_* were defined as the cumulative sum of *T*_1_ and the randomly drawn inter-response intervals. The duration of the go-period was arbitrarily set to 1200 ms. *T_N_* was defined as the last button press before the time-point defined by the sum of stop signal and the simulated *SSRT*. A data set consisting of *N* such simulated trials (*N* = 100, 250, 500, 1000, 2500, 5000, 10000) was then analyzed following the same deconvolution routine used for the actual data. The *SSRT* values were drawn from one of three different ex-Gauss distributions (SSRT1 = ex-Gauss[μ = 190, σ = 0.030, τ = 0.0] sec, SSRT2 = ex-Gauss[μ = 0.168, σ = 0.020, τ = 0.022] sec, SSRT3 = ex-Gauss[μ = 0.212, σ = 0.020, τ = −0.022] sec). For negative values of τ we subtracted the exponential component from the Gaussian component rather than adding it. The values were chosen to yield two skewed and one non-skewed distribution of equal mean (190 ms) and standard deviation (30 ms). Hence any difference in the recovered distribution would be attributable the change in shape of the distribution rather than its position or width.

**Figure 5:**
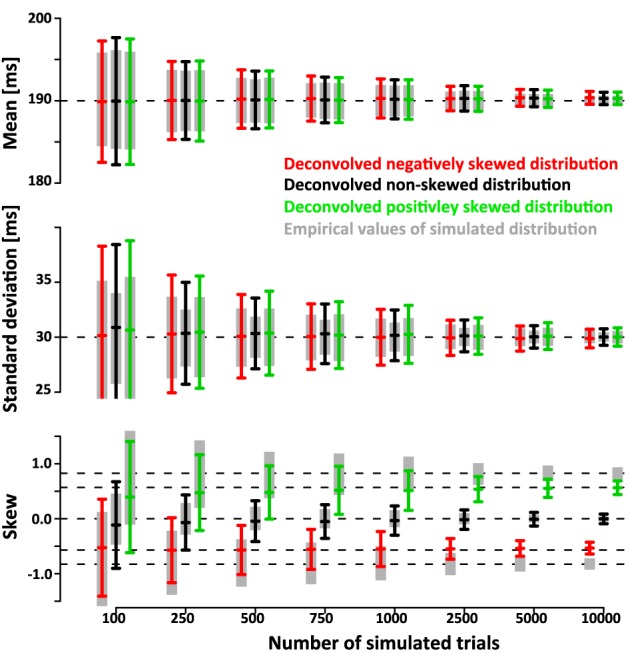
**Ninety five percent confidence intervals (mean ± 1.96×standard deviation) of recovered mean, standard deviation and skew of** ***SSRT*** **as a function of numbers of simulated trials N (1000 simulated data sets per confidence interval)**. The gray bars indicate mean, standard deviation and skew of the simulated *SSRT* distributions that were then used to simulate times of last button presses *T_N_*. The original SSRT distributions were then recovered from the distributions of simulated *T_N_* using the deconvolution algorithm. The black bars indicate the 95% confidence interval for the deconvolved parameters when SSRTs were drawn from a non-skewed Gaussian distribution of mean 190 ms and standard deviation of 30 ms. The red and green bars indicate the corresponding 95% confidence intervals when SSRTs were drawn from positively and negatively skewed ex-Gauss distributions of identical mean and variance. Note that the confidence intervals of the deconvolved distributions are only a small fraction larger than the confidence intervals of the actual empricial distributions. The deconvolution algorithm operates on smoothed *T_N_* distributions (temporal filtering with a 16 ms Gaussian kernel). Hence, the recovered distributions reflect properties of the filtered SSRT distribution. Filtering has no effect on the mean. However, it does affect the standard deviation and the skew. The effect of filtering can easily be removed from the estimate of standard deviation (note that the sd estimates of the deconvolved distributions converge to the true value of 30 ms). A similar approach can be taken to recover true skew, but this operation depends on the assumption that the underlying distributions are ex-Gaussian. To maintain a model-free approach, we did not use such a correction here. As a result, both positive (and negative) skew values are underestimated (see red line). It is important to note, however, that the recovered skew values matche the true skew value of the ex-Gauss that has been filtered with the same temporal kernel that was used in the deconvolution approach.

The simulations show that the deconvolution algorithm accurately recovers the different shapes of the distributions, in this case a left-, non- and right-skewed shape. The accuracy was quantified in two ways. First, we extracted 95% confidence intervals (mean ± 1.96^*^standard deviation) of the estimated mean, standard deviation and skew. Figure 5 shows the confidence intervals as a function of trial number. Even for a relatively low number of ~750 trials the confidence intervals are quite small (less than ±4 ms for mean and standard deviation, less than ±0.4 for skew).

**Figure 6:**
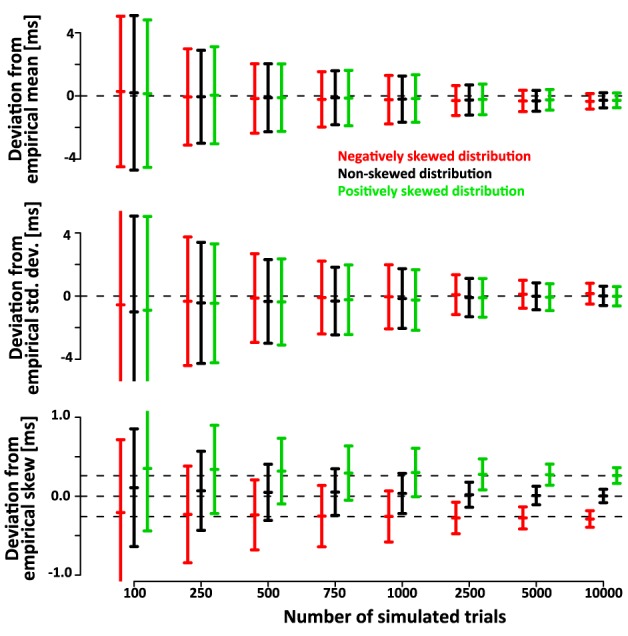
**Mean, std.dev. and skew of the simulated SSRTs varies around the true parameters due to the finite size of the sample**. Hence, the confidence bars in Figure 5 reflect both, the error of the deconvolution algorithm as well as the random sampling of the empirical distribution upon which the deconvolution is based. Here we subtract the recovered parameters of the deconvolved distributions from those of the underlying empirical distribution. The confidence intervals are substantially smaller, indicating that the deconvolution algorithm accurately recovers the variability of the empirical distribution. This is further validated by correlation between the empirical and recovered mean (ρ*_Gauss_* = 0.77, ρ_*ex-Gauss*_ = 0.77), standard deviation (ρ*_Gauss_* = 0.54, ρ*_ex-Gauss_* = 0.66) and skew (ρ*_Gauss_* = 0.37, ρ*_ex-Gauss_* = 0.63). These correlations are non-trivial, because the true mean, standard deviation and skew are constant within the conditions.

Note that the confidence intervals of the parameters of the deconvolved distributions are only a small fraction larger than the confidence intervals of the parameters of the simulated sample. This indicates that a large fraction of the variance is due to variability of the simulated sample around its true parameters rather than errors introduced by the deconvolution algorithm. After subtracting the recovered parameters from those of the underlying simulated sample (rather than the ones of the theoretical distribution) the confidence intervals are approximately half as wide (Figure 6). This indicates that the deconvolution algorithm accurately captures small deviations of the simulated sample from the theoretical distribution they are drawn from.

**Figure 7:**
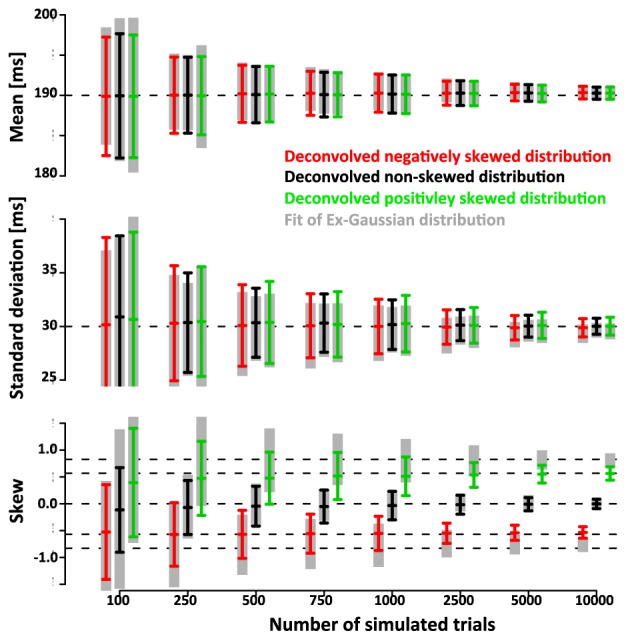
**Comparison of the non-parametric deconvolution algorithm with a paramtric approach based on ex-Gauss distributions**. Note that both approaches provide estimates of very similar accuracy. The main advantage of the ex-Gauss approach is that is allows an unbiased estimation of the skew—the deconvolution algorithm estimates the skew of the smoothed function which is always deviated toward zero. The main advantage of the deconvolution approach is that it does not restrict the range of possible shapes of the distribution—in the current case the simulated distributions were drawn from the same family of distributions that were used in the parametric fit. For real data, the underlying distribution is not known and may not be part of the ex-Gauss family.

We further quantified the accuracy of the deconvolution algorithm by comparing it to the accuracy of a parametric approach based on the fit of an ex-Gauss distribution. Figure 7 shows that the confidence intervals of the parametric approach are highly similar to the ones obtained with the non-parametric approach. It is important to keep in mind that in this case the parametric approach should be particularly effective as it uses the same parametrization that was used to generate simulated data, an advantage that is not necessarily true for real data.

**Figure 8:**
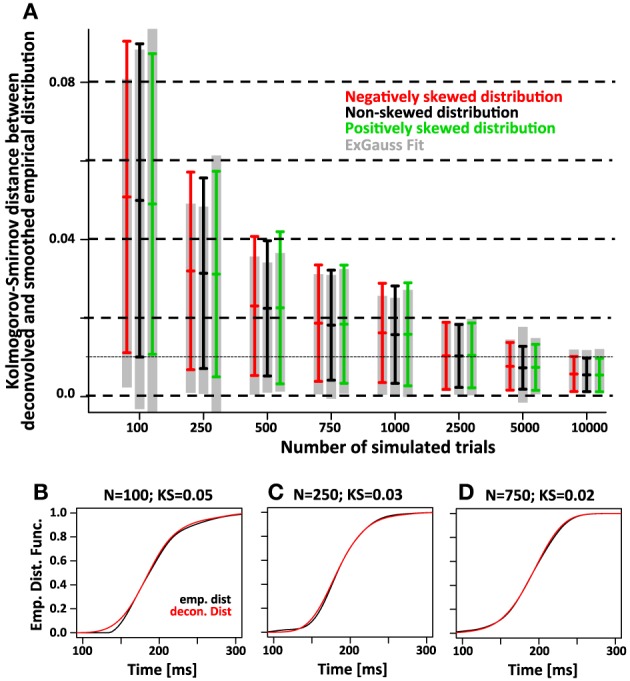
**The accuracy of the deconvolution algorithm measured as the maximum difference between the smoothed empirical distribution function and the deconvolved distribution (Kolmogorov-Smirnov statistic)**. **(A)** As the number of simulated trials increases, the KS statistic between the smoothed empirical distribution and the distribution recovered with the deconvolution algorithm approaches zero. This is true regardless of the skew of the distribution (red, black, and green color). The gray bars indicate the same KS statistic for the distribution that was recovered by fitting the ex-Gauss distribution. **(B–D)** Three examples for individual simulations with representative KS-values for either 100, 250, and 750 trials. The black line indicates the smoothed empirical distribution function, the red line the recovered distribution. The examples were chosen to have a KS statistic of 0.05, 0.03, and 0.02, respectively. Note that for KS values of 0.03 and 0.02, the recovered distribution is almost identical to the original distribution.

Finally we compared the accuracy of the two approaches using the maximum difference between the recovered and the smoothed empirical distribution function (Kolmogorv-Smirnov Statistic, Figure 8). For 750 trials we expect average KS values of the deconvolution approach is ~0.02 and on par with the KS values obtained with the parametric approach.

### SSRT-deconvolution of real data

**Figure 9:**
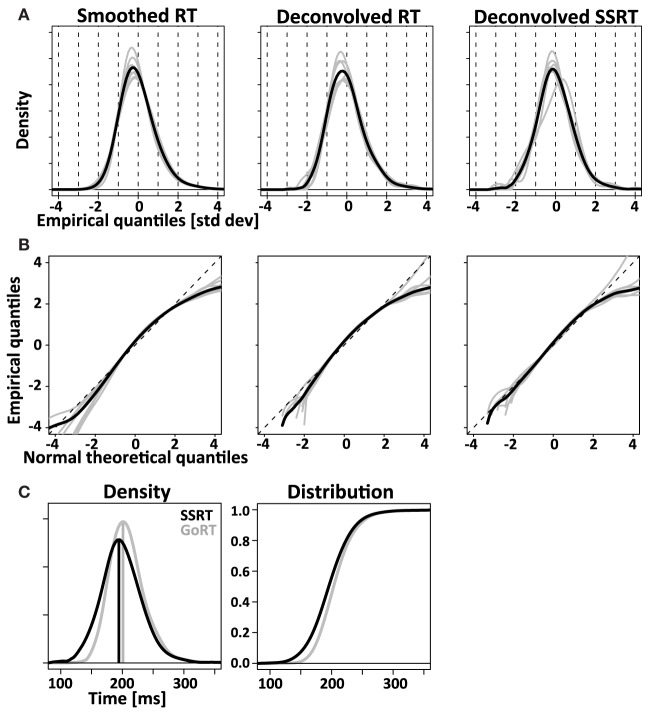
**Comparing RT and SSRT distribution**. Left column: *RT* distributions smoothed with the same Gaussian kernel used for the deconvolution algorithm. Middle column: *RT* distributions after empirical convolution with *X* and subsequent application of the deconvolution algorithm. Right column: deconvolved *SSRT* distributions. Comparison of left and middle column visualize the modest distortions introduced by the deconvolution algorithm. Comparison between middle and right column visualize the difference between response initiation (*RT*) and response inhibition (*SSRT*). **(A)** Normalized (z-transformed) empirical density functions show significant rightward-skew (*t*-test, *p* < 0.05) for all three distributions (smoothed *RT*, deconvolved *RT* and deconvolved *SSRT*). **(B)** Quantile-quantile plots visualize rightward skew as a concave curvature. **(C)** Direct comparison of average GoRT and SSRT density function (left) and distribution function (right). To account for different mean and standard deviation, distributions were z-transformed, averaged and transformed back using average values of mean and standard deviation for SSRT and GoRT, respectively. The initial normalization allows effective averaging that maintains the shape of the distributions despite considerable differences in mean and standard deviation. The back-transformation reintroduces the differences in mean and standard deviation that were lost in the initial normalization step.

To verify the deconvolution approach on real data, we empirically convolved the known GoRT distribution with our estimate of *X* (see Figure 1 and Methods) and used the same deconvolution method to recover the known *GoRT* distribution (see Methods). Figure 9A shows the recovered normalized *GoRT* density function (middle column) as well as the original smoothed and normalized *GoRT* density function (left column). The results highlight to ability of the deconvolution method to recover the original *GoRT* distribution. The recovered *GoRT* densities are noisier, but their specific shape, including the characteristic rightward skew, was accurately recovered. The skew of the recovered *GoRT* distribution is significantly larger than zero (1.1 ± 0.6, *t*-test, *p* < 0.05) and very similar to the skew of smoothed *GoRT* distribution (1.2 ± 0.7).

Using the same approach we deconvolved *T_N_* to obtain an estimate of the *SSRT* density. Similar to the recovered *GoRT* densities, the *SSRT* densities were significantly right-skewed (0.9 ± 0.7, *t*-test, *p* < 0.05). The rightward skew is visible in the recovered densities (Figure 9A) as well as the quantile-quantile plots (Figure 9B).

**Figure 10:**
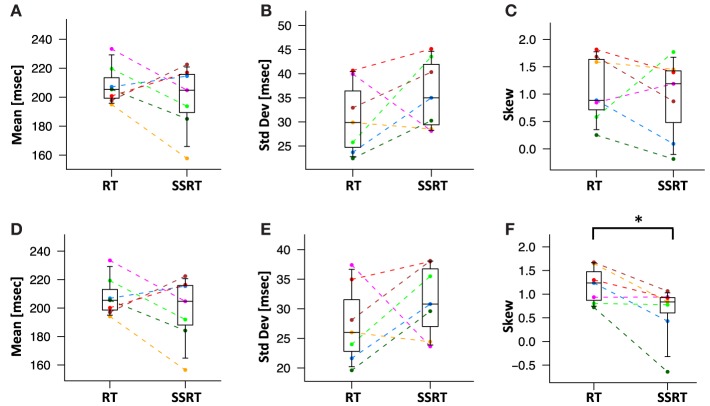
**Mean, standard deviation and skew of deconvolved** ***RT*** **and** ***SSRT*** **distributions**. Different colors represent different subjects. Statistical significance was assessed with two-sided paired *t*-tests (^*^:*p* < 0.05, ^**^:*p* < 0.01). **(A)** Mean of *RT* and *SSRT* are statistically not distinguishable. **(B)** The standard deviation of the *RT* and *SSRT* distributions are not significantly different from each other. **(C)** The skew of the *RT* and *SSRT* distributions are not significantly different from each other. The skew of *RT* and *SSRT* distribution are significantly larger than zero. **(D–F)** Mean, standard deviation and skew from the parametric ex-Gauss fit. The parametric fit confirms the same pattern, i.e., somewhat faster SSRTs with somewhat higher standard deviation and less rightward skew (paired *t*-test, *p* = 0.02).

We then directly compared the first three moments of the recovered *GoRT* and *SSRT* distribution (Figures 10A–C and Table 1). Mean *SSRT* (199 ± 23) is 10 ms shorter than mean *GoRT* (209 ± 15 ms). This difference does not reach significance (paired *t*-test, *df* = 6, *p* = 0.36). Standard deviation of the *SSRT* distribution (38 ± 7 ms) is 5 ms larger than standard deviation of the *GoRT* distribution (33 ± 7 ms). This difference also does not reach significance (paired *t*-test, *df* = 6, *p* = 0.21). Mean skew for the *SSRT* distribution (0.9 ± 0.7) is ~20% lower than the skew of the *GoRT* distribution (1.1 ± 0.6). This difference does not reach significance (paired *t*-test, *df* = 6, *p* = 0.59). Similar results were found when using the parametric ex-Gauss approach (Figures 10D–F and Table 1). In this case, however, the skew for the *SSRT* distributions was significantly smaller than the skew for the *GoRTs* (paired *t*-test, *df* = 6, *p* = 0.02). We followed up on this discrepancy between the deconvolution and ex-Gauss approach and noticed that the difference manifests itself in two subjects. The deconvolved *SSRT* densities of these subjects have mass in a few long time-bins that could be considered outliers. In order to be maximally inclusive, the outlier rejection for the deconvolution approach was very liberal (see Methods). However, the parametric approach had implicitly classified these bins as outliers thus yielding substantially smaller skew estimates for these two subjects. To exclude the impact of these potential outliers we used Bowley's more robust quantile-based skew estimate. For a particular quantile *0* < *u* < *0.5*, the Bowley's skew estimate is defined as:

$$robustSkew\left(u\right)=\frac{F^{-1}\left(u\right)+F^{-1}\left(1-u\right)-2F^{-1}\left(0.5\right)}{F^{-1}\left(u\right)+F^{-1}\left(1-u\right)}$$

**Table 1:** **Mean, standard deviation and skew for GoRT, recovered GoRT and recovered SSRT distributions**.

		**Mean [ms]**	**Std. dev [ms]**	**Skew**
Reaction time	Raw	208 ± 14	29 ± 7	1.5 ± 0.5
	Binned	208 ± 14	29 ± 8	1.7 ± 0.7
	Smoothed	208 ± 14	34 ± 7	1.2 ± 0.7
Recovered reaction time	Deconvolution	208 ±14	33 ± 7	1.1 ± 0.6
	Ex-Gauss Fit	208 ± 14	27 ± 7	1.2 ± 0.4
Recovered SSRT	Deconvolution	199 ± 23	38 ± 7	0.9 ± 0.7
	Ex-Gauss Fit	199 ± 23	31 ± 6	0.6 ± 0.6

*Gray shading indicates conditions that involve either direct or indirect smoothing with a Gaussian kernel of 16 ms. These conditions have by definition larger standard deviations and skew values deviated toward zero*.

Setting *u* to 0.1 yields Kelley's absolute measure of skewness. Using Kelley's quantile-based absolute measure of skewness, we find a significantly smaller skew for the *SSRT* distributions when using the deconvolution approach (paired *t*-test, *df* = 6, *p* = 0.04). This reconciles the findings of the parametric and the non-parametric approaches and identifies smaller positive skew as one of the main differences between *GoRT* and *SSRT* distributions.

The overall picture conveyed by both methods is that of strikingly similar *SSRT* and *GoRT* distributions. However, our results suggest that *SSRTs* are somewhat shorter and have a wider and less skewed shape. This finding is nicely illustrated in Figure 9C that directly compares position and shape of the averaged *SSRT* and *GoRT* distributions side by side. Note that the main difference is the earlier rise of the ascending flank of the *SSRT* density function. In contrast, the falling flanks of the two density functions almost overlay.

## Discussion

Response inhibition is a critical aspect of motor and cognitive control, and is thought to involve prefrontal cortex and basal ganglia; specifically, the hyperdirect cortico-striatal pathway. Using a small sample of young healthy control subjects trained on the task, the current study showcases the feasibility of the non-parametric method to estimate entire *SSRT* distributions from ongoing motor sequences with discrete behavioral output such as typing. SSRT distributions allow us to study the fine-scale functional properties of the neural pathways that mediate inhibitory control with high temporal resolution. The non-parametric nature of the approach is particularly important and appealing as it complements a recently developed parametric approach (Matzke et al., 2013). Previous non-parametric approaches have never been implemented because they were estimated to depend on a prohibitively large number of ~250,000 stop signal trials per stop signal delay (SSD) for a total number of ~1,000,000 trials for each stop-signal delay to be used in the experiment (Matzke et al., 2013). Our simulations show that the current deconvolution approach yields adequate estimates of entire *SSRT* distributions (KS statistic ~0.02) for as little as 750 trials.

This reduction in the number of required trials derives from the specific design of the SeqIn task. Rather than using a single discrete motor act, it uses a quasi-continuous motor sequence. Hence, our approach is related to an SSRT-paradigm developed by Morein-Zamir and colleagues (the *continuous tracking task*), where subjects continuously exert pressure with their index finger until a stop signal instructs them to stop pressing (Morein-Zamir et al., 2004, 2006a,b). The *continuous tracking task* has the advantage that each trial provides one explicit estimate of SSRT and hence has a higher information content than a trial in the countermanding task. However, Morein-Zamir and colleagues also acknowledge one potential criticism of the *continuous tracking task*, namely that in this task stopping might be considered an action (*“pull finger upwards”*) rather than the inhibition of an action (*“stop pushing finger downwards”*) (Morein-Zamir et al., 2004). Our task addresses this potential issue by using a dynamic motor sequence that clearly needs to be inhibited when the stop-signal occurs. At the same time, it maintains the advantage of higher information content: on each trial SSRT was too slow to inhibit the last observed button press (*SSRT* > *T_N_*), yet fast enough to inhibit the next button press (*SSRT* < *T*_*N*+1_). Because on average, button presses occur once every ~30 ms, each trial narrows down SSRT to a window of ~30 ms. This is substantially more information than available from individual stop-signal trials of the standard SSRT task: SSRTs are either longer (failed inhibition) or shorter (successful inhibition) than a particular value (determined by SSD and mean RT), and hence the information content is to a first approximation binary.

A second reason for the substantial reduction in the number of trials is that in the SeqIn task every trial is a stop-signal trial that directly contributes to the estimation of *SSRT*. This is in contrast to countermanding tasks where only 20–25% of trials have a stop signal. It is possible to include 100% stop trials in the SeqIn task because the timing of the stop-signal within each trial is unpredictable over a range of 3.5 s. However, in terms of experimental duration (rather than trial count), the benefit of having a stop-signal on each trial is partially countered by the fact that each individual trial in the SeqIn task is longer than in countermanding paradigms. Nevertheless, it is still significantly faster to collect 750 trials of the SeqIn task, than 1,000,000 trials of a countermanding paradigm.

The third reason that leads to the reduction of the number of trials is that the deconvolution approach applies temporal smoothing to the distributions of the last button presses *T_N_* prior to the deconvolution. Smoothing reduces the number of trials that are needed to recover meaningful *SSRT* distributions. Otherwise, smoothing is not critical because it maintains the main features of the distribution that are typically present in lower frequency bands. In fact, even regular reaction-time distributions are often visualized using either implicit or explicit smoothing. The effects of smoothing on the shape of the distribution are known and can be taken into account either quantitatively (standard deviation) or at least qualitatively (skew).

Here we used the SeqIn task to recover entire *SSRT* distributions for the inhibition of an unconstrained ongoing motor sequence (finger tapping). We also measured the *GoRT* distributions for the initiation of the same motor sequence. We find that in the SeqIn task, mean *GoRT* and mean *SSRT* are statistically indistinguishable (*GoRT*: 208 ± 14 ms; SSRT: 199 ± 23 ms). The finding of similar values for the two tasks may seem somewhat surprising because in most other studies mean *RT*s are 100–200 ms slower than mean *SSRT*s. However, this apparent discrepancy is due to the fact that most other paradigms measure choice *RT*s whereas the current paradigm measures “simple” *RT*s without a choice component. Because *SSRT* itself does not have a choice component, the current approach enables a fair comparison between *GoRT* and *SSRT*. Furthermore, the SeqIn task was specifically designed to facilitate the comparison between *GoRT* and *SSRT*: the Go- and Stop signals themselves are identical audio-visual events, and waiting time distributions for the Stop- and Go signal are identical thus equalizing any anticipation/prediction for both signals. After thus equating the conditions for the two responses, mean latency of response initiation is almost identical to the mean latency of the response inhibition. Note that we are not claiming statistical equality of latency (or width). In fact, we expect that larger sample size will reveal significantly shorter SSRT latencies. We merely point out that the absolute differences between the GoRT and SSRT distributions (significant or not) will be small relative their variability. This suggests largely similar (but not necessarily identical) functional properties of the two neural pathways that mediate response inhibition and response initiation.

While both *SSRT* and *GoRT* have statistically indistinguishable mean latency and width in this small sample, *SSRT* distributions have significantly smaller skew. This is particularly interesting, because it suggests that while GoRT and SSRT may be strikingly similar at first glance, there may be subtle yet meaningful differences. Once confirmed in a larger sample, these subtle differences can be used to test different mechanistic models of inhibitory control such as the independent race model (Boucher et al., 2007) or the Hanes-Carpenter model (Hanes and Carpenter, 1999) or the so-called special race model (Logan et al., 2014). The differences in the shape of *SSRT* could in principle be mapped to parameters of these models, which in turn may map onto parts of the direct and hyperdirect cortico-striatal pathways. Such a mechanistic and parametric approach will allow us to address a number of interesting questions: Are the drift rates of the Go and Stop process identical? Are the heights of the response- and the inhibition bound identical? Can the same types of model that successfully explain *GoRT* distributions provide a satisfactory fit for *SSRT*? Can *SSRTs* be explained in terms of a single mechanism, or do we need to consider more complex dual-process accounts? Some of these questions have already begun to be explored in recent studies by Matzke et al. (2013) and Logan et al. (2014). Because such approaches necessarily depend on various parametrizations of the *SSRT* distribution, it is particularly important to validate the shape of parametrically recovered distributions with non-parametric methods.

At this point we want to provide a brief comparison of our non-parametrically recovered SSRT distributions with the parametrically recovered ones from the study of Matzke et al. (2013). Overall, the mean SSRTs in our task (~200 ms) were somewhat shorter than the ones found by Matzke and colleagues (~220–230 ms). This difference can most likely be attributed to our sample that was comprised of young, highly motivated and experienced psychophysics subjects compared to a more representative sample examined by Matzke and colleagues. However, it is important to note that the single-subject range of SSRTs in our sample is well within the range of SSRTs of Matzke's and other SSRT studies. The shape of the SSRT distribution in our study was narrower and less skewed. To quantify this difference we compared our mean values of the parameters of the ex-Gaussian distributions in our study to the ones estimated from Figure 17 in the paper by Matzke (mean of Gaussian component: 185 vs. ~160 ms; standard deviation of Gaussian component: 23 vs. ~20 ms, and lastly the exponential component: 20 vs. ~60 ms). Hence, the biggest difference between the two studies is the exponential component that plays a much bigger role in the data by Matzke. It is important to note the large inter-individual variability of SSRT distributions in the sample by Matzke. Some of the subjects have very narrow and barely skewed SSRT distributions, very much like the subjects in our sample. Hence, it is possible that the overall difference of SSRT distributions resembles the stricter selection criterion of subjects in our study. However, it is also important to note that Matzke observed a strong dependence of SSRT distribution on the fraction of stop-signal trials. A data set with 20% stop trials had significantly wider and more right-skewed distribution compared to a data set with 40% stop trials. While our paradigm is somewhat different it has a stop-signal in 100% of the trials. Hence, some of the differences may also be attributed to the higher ratio of trials with a stop-signal.

Any estimate of SSRT or GoRT distributions implicitly assumes that the variable in question is stationary during the data-acquisition period. Hence, we tested if there is any indication of systematic changes in GoRT and SSRT over the course of the experiment. Our data indicated that GoRT and SSRT stayed constant over the course of the experiment. This suggests that the subjects had enough training and were performing at ceiling levels during the entire experiment. However, we also tested whether performance stayed constant within each behavioral session consisting of 3 blocks of 25 trials. We observed a significant increase of SSRT over the course of each behavioral session. GoRTs in contrast, remained stable. This systematic SSRT slow-down affects the comparison between GoRT and SSRT distributions that is based on all of the data, including blocks where SSRTs have already slowed down. In particular, it may have led to an overestimation of mean SSRT and the width of the SSRT distribution. In addition it may have led to an underestimation of the skew of SSRT.

The SSRT slowdown was an incidental finding outside of the main focus of the study that was aimed at exploring technical feasibility of the deconvolution algorithm. It is not our intent to draw conclusions about SSRT slowdown form the small sample of subjects. Nevertheless, the finding was intriguing enough to warrant some speculation about its potential origin. In particular we want to rule out two trivial explanations. (1) The observed SSRT slowdown can not be explained by a reduction of attention or arousal. If so, we would also expect a corresponding reduction of GoRT. (2) SSRT slowdown cannot be explained by a tradeoff in the balance between going and stopping: first, the minor reduction of GoRT does not seem to be consistent with the substantially larger increase of SSRT. Second, the SeqIn task is not a dual task (as the countermanding task) where subjects need to prioritize either one or the other of the tasks.

We want to end the discussion by addressing certain limitations and anticipating potential criticisms. (1) The analysis and interpretation of our data depends on the assumption of independence between the Go and Stop process. Our study did not allow us to explicitly test this assumption. However, the assumption of independence is central not only to our paradigm, but also to all other independent race-models. Studies using countermanding tasks have (a) indicated that violations of independence are moderate, and (b) indicated that SSRT measured in countermanding tasks are reasonably robust against violations of the assumption of independence. Future studies will be necessary to test if the same is true for the SeqIn task.

(2) The overwhelming majority of studies of inhibitory control use the countermanding paradigm in which the to-be-inhibited response is the result of a binary decision process. This type of task has been extremely useful to study response inhibition in healthy controls, and response inhibition deficits in a number of neuro-psychiatric conditions. However, the concept of a Stop process and SSRT, have been formulated independent of this particular paradigm. In fact, other paradigms have been developed in the past, and were shown to correlate with SSRT measured in the countermanding paradigm. Hence, while terms like “SSRT” and “Stop process” have been intimately linked with the countermanding paradigm, their use in the current context is very much within the original definition that does not specify that the to-be-inhibited action must be the result of a binary decision process. Nevertheless, we want to caution that the SeqIn task should not be used as a substitute for established countermanding paradigms such as the Stop-It, SST or Vink task until it has formally been shown to measure the same construct. However, based on the similarity of our task with the complex motion task by Logan and the continuous tracking task by Morein-Zamir both of which are believed to measure the same construct we are confident that a validation study will confirm that the SeqIn task measures the same construct.

(3) As pointed out above, it is not yet 100% clear if the SeqIn task measures the same variant of inhibitory control as countermanding tasks. On the flipside of this argument, it is not clear if countermanding paradigms measure the same construct of inhibitory control that is involved in stopping ongoing motor sequences. Situations in which an ongoing motor sequence needs to be inhibited are prevalent in real life and constitute an important area of study. For example, a quarterback may need to abort a particular play immediately after the snap, right before the ball leaves his hand, or at any point during the execution of the complex motor sequence that takes place between the snap and the pass. In fact, even the standard example of inhibitory control—a baseball player aborting a swing at a ball outside the strike-zone—arguably shares more similarity with the SeqIn than the countermanding task. Similarly, many situations of inhibitory control of relevance in neuropsychiatric conditions require the interruption of an ongoing motor sequence, such as the interruption of perseverative hand washing in obsessive compulsive disorder. In the most likely scenario, the two types of inhibitory control involve identical neural mechanisms and insight from one type of task will be relevant to both types of scenario. However, it is also theoretically possible that different neural mechanisms are involved in the two tasks and that insights from standard SSRT tasks do not extrapolate to the inhibition of ongoing motor sequences. In this case the SeqIn task will be one of only very few tasks to measure inhibitory control of ongoing motor sequences.

(4) Based on the fact that SSRT and GoRT have similar means (~200 ms), it has been argued by some that the motor sequence in the SeqIn task may be stopped without the need to engage inhibitory control. Rather than inhibiting the ongoing motor sequence it might be sufficient to just refrain from issuing additional motor commands, or issue a new motor command (“*pull all fingers upwards*”) that will override other motor commands and prevent additional finger taps. However, this assumption cannot account for the selective slow-down of SSRT across a session while RT remains stable. Also, it is important, albeit less obvious, to note that the same argument can be used to question the involvement of inhibitory control in the countermanding task: While it may take on average 400–600 ms to press the correct button in the choice task, it will still take only ~200 ms to issue a simple “*pull-all-fingers-upwards*” motor command in response to the onset of the stop-signal. In fact, it has been argued that even in the countermanding task an explicit stop process is not needed to explain the behavioral findings (Salinas and Stanford, 2013). Hence, the selective SSRT slow-down in our task may be one important piece of *behavioral* data that supports the presence of an inhibitory process with distinct properties. In summary, these arguments clearly refute the idea that the duration of the GoRT relative to SSRT has any influence on whether or not the stopping of the motor sequence needs to involve inhibitory control. However, we also want to acknowledge that at this point, the SeqIn task has not been performed in the context of fMRI or single-cell recordings. Hence, there is currently no neural evidence in favor or against the notion that the SeqIn task engages the hyper-direct pathway of the basal ganglia.

## Funding

This work was supported by National Institutes of Health Grant MH059244 (V.P.F.) and German Research Foundation Grant TE819/1-1 (T.T.).

### Conflict of interest statement

The authors declare that the research was conducted in the absence of any commercial or financial relationships that could be construed as a potential conflict of interest.
